# Rare Signet Ring Cell Adenocarcinoma of the Colon Metastasis to the Orbit

**DOI:** 10.1155/2020/2940579

**Published:** 2020-02-25

**Authors:** Colin Bacorn, Lily Koo Lin

**Affiliations:** Department of Ophthalmology and Vision Science, University of California Davis Health, Sacramento CA, USA

## Abstract

Metastases arising from primary tumors of the gastrointestinal tract are not commonly encountered in the orbit. Colorectal carcinomas are subcategorized based on morphological and genetic characteristics with these distinctions bearing therapeutic and prognostic significance. The behavior of these subcategories, including their propensity for orbital metastasis, differs, and clinicians treating these tumors must be aware of their metastatic profiles. This report describes a 51-year-old female with right upper lid swelling and ptosis ultimately found, what we believe to be, the first reported case of signet ring cell colon carcinoma metastasizing to the levator muscle and superior orbit. This case serves as a reminder to all clinicians to consider orbital metastasis even in malignancies not typically found in this location.

## 1. Introduction

In the largest case series of orbital tumors to date, 91 of 1264 (7%) were metastatic [1]. The most common primary carcinomas that metastasize to the orbit are breast, prostate, and lung [2]. Orbital metastases carry a poor prognosis with an overall mean survival of 15 months [2]. Metastases from the gastrointestinal tract are very rare with only four reported cases in the Shields series [1]. In this light, it is understandable that, while primary mucinous tumors of the periorbital region have been described, there are very few reports of mucinous or signet ring cell carcinomas metastatic to the orbit from gastrointestinal primaries.

## 2. Case Presentation

A 51-year-old female initially presented with a complaint of abdominal pain and was found to have a large, obstructing, transverse colon mass. Biopsy of this lesion was consistent with a signet ring cell carcinoma. Further work up revealed biopsy-proven metastases to the retroperitoneal lymph nodes and cervix. The patient noted right upper eyelid swelling one month after the diagnosis of her colon cancer, but this was not thought to be related to her malignancy and was not initially investigated further. She underwent ileocolostomy bypass and was treated with a combination of folinic acid, fluorouracil, oxaliplatin (FOLFOX), and cetuximab with initial radiographic response.

**Figure 1:**
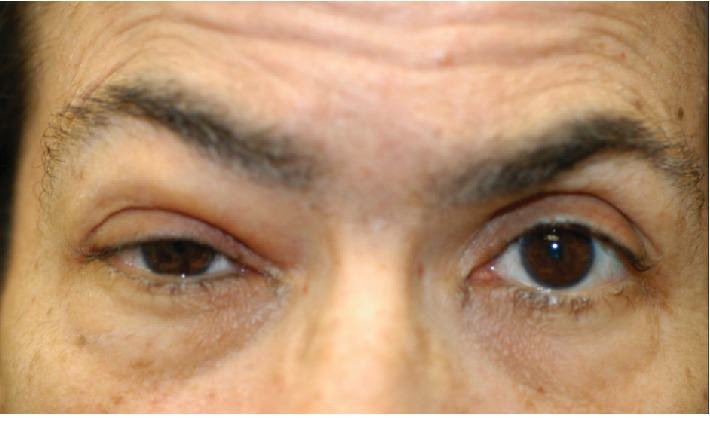
External photograph of the patient's clinical appearance on presentation demonstrating right upper eyelid fullness and ptosis.

Eight months later, the patient presented to the oculoplastics service for evaluation of her eyelid swelling. The lid swelling was painless and had not increased in size since onset. Visual acuity was 20/25 and 20/20. Pupils were without anisocoria or relative afferent pupillary defect. Extraocular motility was full in both eyes. Hertel exophthalmometry measurements at a base of 94 mm were 16.5 mm and 16 mm for the right and left eyes, respectively. Her palpebral fissure widths were 4 mm and 9 mm with decreased levator function of 4-5 mm on the right and normal levator function of 13 mm on the left. There was compensatory frontalis overaction. Margin-to-reflex distances were 0 mm and 3 mm. Right upper eyelid fullness with a rubbery mass of the medial lid was appreciated (Figure 1). The remainder of the ophthalmic examination was unremarkable.

**Figure 2:**
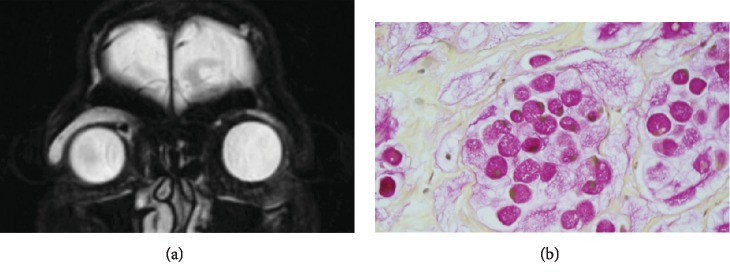
(a) Coronal T2-weighted fat-saturated MR images show a hyperintense mass along the right superior orbit that involves the levator complex and lacrimal gland. (b) Photomicrograph (original magnification, ×60) of the patient's orbital biopsy specimen showing signet ring cells strongly positive for mucicarmine stain (pink).

Given the patient's history of malignancy, she underwent magnetic resonance imaging of the orbits (Figure 2(a)) which revealed a lesion along the superior aspect of the orbit which had not been noted on surveillance imaging from one-year prior. She then underwent anterior orbitotomy for biopsy. Intraoperatively abnormal tissue was noted to infiltrate the levator muscle. Histopathology revealed dermal signet ring cells in a mucinous background (mucicarmine, CK-20, villin, CDX2 positive; CK-7 negative) consistent with metastatic signet ring cell carcinoma (Figure 2(b)). Immunohistochemistry of the lesion was very similar to that observed for the primary colon lesion (mucicarmine, CK-20 positive; villin, CDX2, CK-7 negative). The patient continued her chemotherapy regimen but eventually expired within six months of diagnosis of the orbital metastasis.

## 3. Discussion

Despite colorectal carcinoma being amongst the most common malignancies in the US, ocular and orbital metastases are extremely rare [3–5]. The World Health Organization (WHO) classification of carcinomas of the colon and rectum subcategorizes these malignancies based on histologic appearance with signet ring cell carcinoma (>50% signet ring cells) distinguished from mucinous adenocarcinoma [6]. This distinction has clinical significance as signet ring cell carcinoma is believed to be more aggressive with a poorer prognosis [7–9]. Furthermore, the primary tumor localization (i.e., right colon, left colon, or rectum) also has an impact on prognosis [10, 11].

In the rare instances of metastasis to the eye or orbit, there is a predilection for the choroid with involvement in 80% of cases [12]. However, the tumors reported by Khawaja et al. arose exclusively from colorectal adenocarcinomas and none of the cases were signet ring cell carcinomas. Orbital metastasis of other subtypes of colorectal carcinomas has also been observed [13–15]. Signet ring cell tumors are an uncommon tumor subtype comprising between 0.1% and 2.4% of all colorectal carcinomas [16]. Their typical metastatic pattern is peritoneal carcinomatosis, although atypical spread through hematogenous and lymphatic pathways has also been described [17]. Review of the literature demonstrates very few reports of mucinous and signet ring cell carcinomas metastatic to the orbit from gastrointestinal primaries (three reports of each tumor type). In particular, no other case of signet ring cell carcinoma metastasizing to the orbit from a colon primary was identified [6, 18–22].

Primary mucinous tumors of the periorbital region have also been described. Primary mucinous tumors of the eyelids are relatively indolent and have an incidence on the order of 0.7 per million [23–26]. As both metastatic and primary mucinous tumors can present in the orbital/periorbital region distinguishing between these rare tumors can be challenging [27]. Primary mucinous tumors typically demonstrate absence of CK-20, in contrast with this patient's tumor, and this marker may aid in distinguishing primary mucinous carcinoma from metastasis [28]. Local excision via Mohs micrographic surgery may be appropriate for many cases of primary mucinous eyelid tumors but the prognosis for metastatic colorectal carcinoma is dismal, and treatment with either curative or palliative intent is typically systemic chemotherapy [29, 30].

Although a rare occurrence, clinicians must maintain a high index of suspicion for orbital metastasis in patients with orbital findings and a history of malignancy.
